# Supplementary material for: Improving formaldehyde consumption drives methanol assimilation in engineered E. coli
Source: Nat Commun. 2018 Jun 19;9:2387. doi: 10.1038/s41467-018-04795-4 (PMC6008399; doi:10.1038/s41467-018-04795-4)
Supplement: Supplementary file 1 — Supplementary Information [file 41467_2018_4795_MOESM1_ESM.docx]

**Improving formaldehyde consumption drives methanol assimilation in engineered *E. coli***

Woolston et al.

**Supplementary Note 1**

**Correction of Mdh Δ*G* for Temperature**

Understanding the effect of temperature on the free energy change *ΔG^0^* of NAD-dependent methanol oxidation requires values for the entropy (Δ*S*^0^) and enthalpy (Δ*H*^0^) changes of the reaction. To determine these values, here we use Hess's law in combination with the known heats of formation of methanol, formaldehyde, and hydrogen, and the known Δ*H*^0^ of the H_2_-dependent reduction of NAD^+^, to first calculate Δ*H*^0^ for the MDH reaction. We then calculate Δ*S*^0^ from the estimated Δ*G*^0^ and the relationship $\Delta G^{0}=\Delta H^{0}-T\Delta S^{0}$

According to ^4^, the Δ*H*^0^ of the reaction NAD^+^ + H_2_ 🡪 NADH + H^+^ is -29.2 kJ mol^-1^.

To calculate the enthalpy change of the reaction CH_3_OH 🡪 CH_2_O + H_2_, we use the heats of formation (*H*_f_) in **Supplementary Table 1**, and the relationship

$$\boldsymbol{\Delta}H^{0}=\sum H_{f_{products}}-\sum H_{f_{reactants}}$$

to determine a value of 129.0 kJ mol^-1^. By Hess’s law, the enthalpy change of the reaction

CH_3_OH + NAD^+^ 🡪 CH_2_O + NADH + H^+^ **(1)**

is 99.8 kJ mol^-1^. Using this value, the relationship $\Delta G^{0}=\Delta H^{0}-T\Delta S^{0}$, the temperature (298K), and Δ*G*^0^ = 34.2 kJ mol^-1^, we calculate Δ*S*^0^ = 0.22 kJ mol^-1^ K^-1^. With these values for enthalpy and entropy, and the reasonably assumption that they do not vary significantly in the temperature range of interest, we calculate the temperature-specific values for Δ*G*^0^ used in **Supplementary Figure 1**.

**Supplementary Note 2**

**Sequences of Codon Optimized Genes**

***mdh2 v. 4-1* from C. necator** ^1^

**ATG**ACCCACCTGAACATCGCTAATCGCGTCGACAGCTTCTTCATTCCCTGCGTGACCCTCTTCGGTCCGGGCTGCGTTCGCGAAACGGGCGTGCGCGCCAGATCACTCGGGGCCAGGAAGGCTCTCATCGTCACGGATGCAGGCTTGCACAAGATGGGGCTCTCCGAAGTCGTCGCGGGGCACATTCGCGAAGCCGGGCTCCAGGCCGTCATCTTTCCGGGTGCCGAGCCCAATCCCACCGACGTTAACGTTCACGACGGCGTCAAGTTGTTCGAGCGGGAAGAATGCGACTTCATCGTTTCGCTCGGCGGCGGCTCATCGCACGACTGCGCGAAAGGCATCGGCCTCGTTACCGCCGGAGGCGGACATATCCGCGACTACGAAGGCATCGACAAATCAACGGTGCCAATGACGCCGCTGATTTCGATCAACACGACCGCTGGCACTGCTGCGGAAATGACACGCTTTTGCATCATCACTAATTCGAGCAATCATGTGAAGATGGTGATCGTCGACTGGCGTTGCACGCCATTAATCGCCATCGACGATCCGAGCCTGATGGTCGCGATGCCGCCCGCCTTGACGGCGGCGACCGGCATGGACGCGTTGACTCACGCCATCGAGGCATACGTTTCCACCGCCGCCACGCCAATTACCGATGCCTGTGCGGAGAAGGCGATCGTGCTGATCGCCGAATGGCTGCCCAAAGCTGTCGCGAACGGGGACTCGATGGAAGCACGCGCGGCCATGTGCTACGCCCAATACCTTGCCGGCATGGCCTTCAACAACGCATCACTCGGTTACGTGCACGCGATGGCCCATCAACTCGGCGGCTTCTACAATTTGCCCCACGGCGTGTGCAACGCGATCCTGCTGCCGCACGTGTCGGAATTCAACCTCATTGCCGCGCCGGAGCGCTACGCGAGAATCGCCGAACTGCTAGGCGAGAACATTGGGGGCTTGAGCGCGCATGACGCCGCCAAAGCTGCCGTCTCGGCGATCCGGACCCTTTCCACGTCGATTGGCATTCCGGCGGGTCTGGCGGGCCTGGGCGTCAAGGCGGACGACCATGAAGTGATGGCAAGCAATGCGCAAAAGGATGCTTGCATGCTGACGAATCCGCGCAAGGCCACGCTGGCGCAAGTCATGGCAATCTTCGCTGCGGCGATG**TAA**

***hps* from *B. methanolicus***^2^

**ATG**GAATTGCAATTAGCCTTAGATTTAGTAAACATCGAAGAGGCAAAGCAGGTCGTAGCGGAAGTGCAGGAATACGTCGATATTGTCGAAATCGGGACACCTGTAATCAAAATCTGGGGCTTACAGGCAGTCAAAGCCGTAAAGGATGCCTTCCCACACCTGCAAGTTTTGGCAGATATGAAGACGATGGATGCCGCCGCTTACGAGGTCGCCAAGGCAGCTGAGCATGGTGCTGACATCGTAACAATCTTAGCAGCTGCTGAGGACGTTTCAATCAAAGGCGCCGTAGAGGAAGCCAAGAAACTGGGGAAGAAGATTCTTGTGGATATGATCGCAGTCAAGAATCTGGAGGAACGTGCCAAGCAGGTGGATGAAATGGGCGTTGATTACATTTGCGTCCATGCGGGATACGATTTACAGGCGGTCGGTAAGAACCCACTTGACGATTTAAAACGTATTAAAGCGGTGGTGAAGAACGCGAAGACGGCTATCGCCGGAGGCATTAAGTTAGAAACGCTTCCTGAAGTAATCAAAGCTGAGCCAGATTTAGTAATTGTCGGAGGAGGTATTGCAAACCAAACCGATAAGAAGGCTGCAGCAGAGAAGATTAATAAACTTGTAAAACAAGGTCTGTAAGGATCCAGGAGGAAT**TAA**

***phi* from *M. capsulatus***^3^

**ATG**CATCAGAAACTCATTATCGATAAAATCTCCGGTATCCTGGCCGCGACTGATGCTGGCTACGACGCTAAACTCACTGCTATGCTGGATCAGGCATCACGCATCTTTGTGGCGGGGGCAGGACGTTCTGGGCTGGTGGCTAAATTTTTTGCCATGCGTCTGATGCATGGCGGGTATGACGTCTTCGTAGTCGGCGAGATTGTTACTCCTAGCATCCGTAAAGGCGACCTGCTGATTGTAATTAGCGGTAGTGGCGAGACTGAGACCATGCTTGCGTTTACCAAAAAAGCTAAGGAGCAGGGGGCCTCAATCGCCCTGATTAGTACTCGTGATTCGTCCAGCCTGGGGGATTTGGCAGACTCAGTCTTCCGTATCGGTTCGCCCGAGCTCTTTGGTAAGGTTGTTGGCATGCCTATGGGTACGGTATTCGAACTGAGCACACTTCTGTTTTTAGAAGCAACCATCAGTCATATCATTCATGAAAAAGGTATCCCGGAAGAAGAGATGCGCACCCGTCACGCTAATTTGGAA**TGA**

**Supplementary Note 3**

**Code for Calculating Mdh Michaelis-Menten Parameters**

function michaelis_est

%Takes enzyme activity data across a range of substrate concentrations and

%returns Vmax and Km parameters, along with associated error.

%Author: Ben Woolston

%Date Created: 2017-07-27

%Date Modified: 2018-04-04

clc

format shortEng

%X is a vector of methanol concentrations (in mM) used for activity

%measurements

X = [0

10

25

50

100

200

350

500

750

1000

0

10

25

50

100

200

350

500

750

1000

0

10

25

50

100

200

350

500];

%Y is a vector of Mdh activity mesurements corresponding to the methanol

%concentrations in X

Y = [0

11.23428571

21.64571429

39.06285714

56.30857143

78.35428571

95.39428571

111.8971429

123.6

114.2914286

0.651428571

10.50285714

22.88

38.03428571

53.80571429

78.20571429

99.10857143

112.7771429

114.3085714

112.8

0.822857143

9.92

19.32571429

34.72

51.26857143

72.24

89.61142857

106.4571429];

%Parameters: b(1) = vmax, b(2) = Km

mm = @(b,x)b(1)*x./(b(2)+x); %Define the Michaelis-Menten relationship

beta0 = [87, 132]; %Initial Michaelis-Menten paramaeter guesses

[beta,r,j,cov,mse] = nlinfit(X,Y,mm,beta0) %Calculate best-fit VMax and Km parameters from measured activity data

ci = nlparci(beta,r,'Jacobian',j) %Calculate a confidence interval around Michaelis-Menten parameters from the nonlinear fit in the previous line

end

**
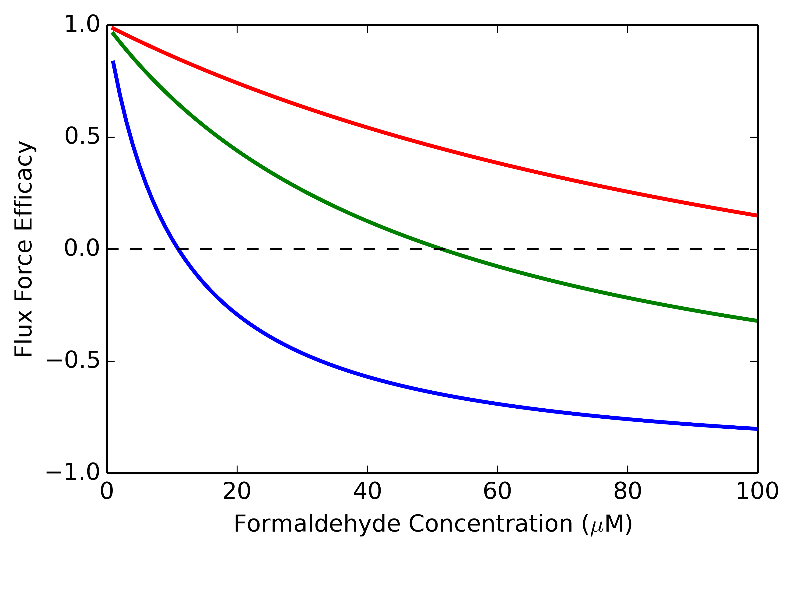
**

**Supplementary Figure 1. Flux-force efficacy for Mdh reaction**

The flux-force-efficacy (FFE)^8^ was calculated as a function of formaldehyde concentration for a typical NAD:NADH ratio in growing cells, for three different temperatures: 25°C (Blue), 37°C (Optimal *E. coli* growth, Green), and 45°C (Optimal *B. methanolicus* growth, Red). NAD and NADH concentrations were 2.6 mM and 0.083 mM, respectively^9^. ΔG^0^ was taken from the Equilibrator database^10^, and corrected for temperature as described in **Supplementary Note 2**.

**
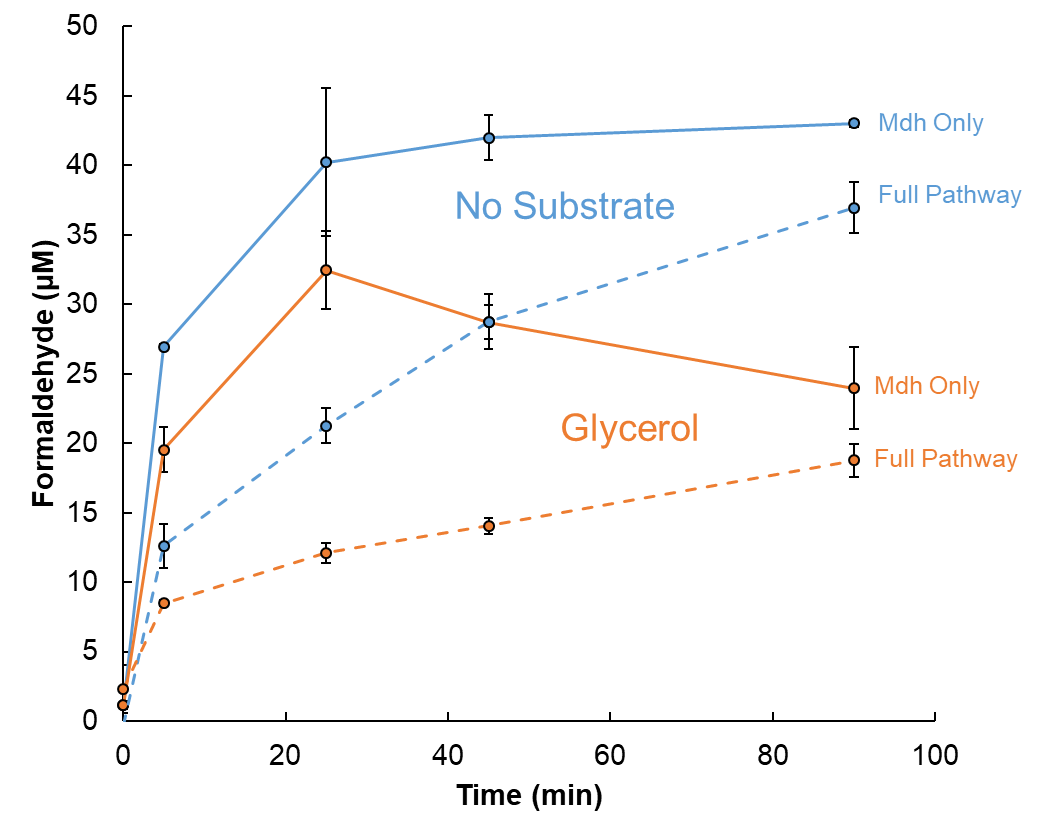
**

**Supplementary Figure 2. Glycerol does not elicit the same phenotype as xylose**

Formaldehyde levels over time after addition of 250 mM methanol to starved cells of *E. coli* MG1655(DE3) Δ*frmA* with either no additional substrate (blue) or 4 g/L glycerol (orange). Solid lines represent cells expressing only Mdh, and dashed lines denote cells expressing the full methanol assimilation pathway (Mdh, Hps and Phi). Error bars represent s.d. of n = 3 biological replicates (three individual colonies).

**
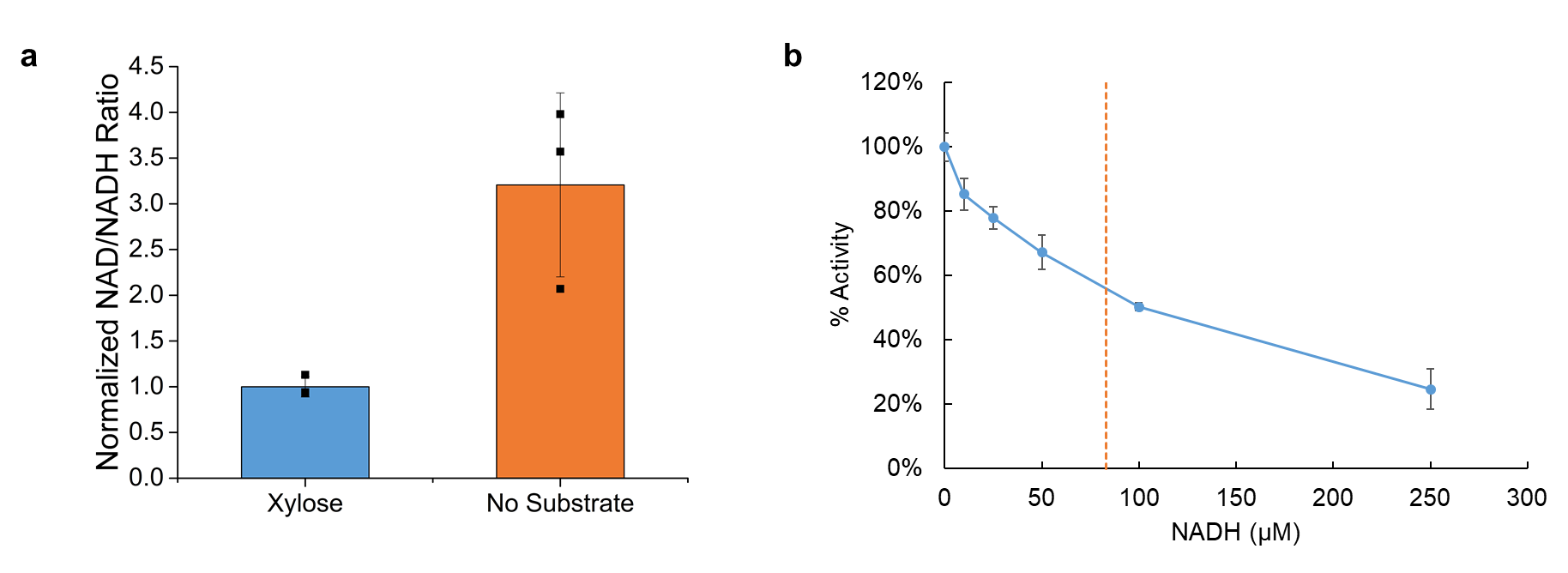
**

**Supplementary Figure 3. NAD/NADH ratios and effect of NADH on Mdh activity**

**a)** NAD and NADH concentrations in resting cells of *E. coli* MG1655(DE3) *ΔfrmA* with either no substrate or 6 g L^-1^ xylose were quantified by LC-MS/MS as described in the methods sections, and the ratios normalized to the xylose case. Error bars represent s.d. of n = three biological replicates (individual colonies). **b)** Effect of NADH on initial velocity of purified Mdh. Velocities are normalized to no-NADH control, and the orange line signifies the intracellular concentration of NADH from Bennet et al., 2009^9^. Error bars represent s.d. from n = three technical replicates.


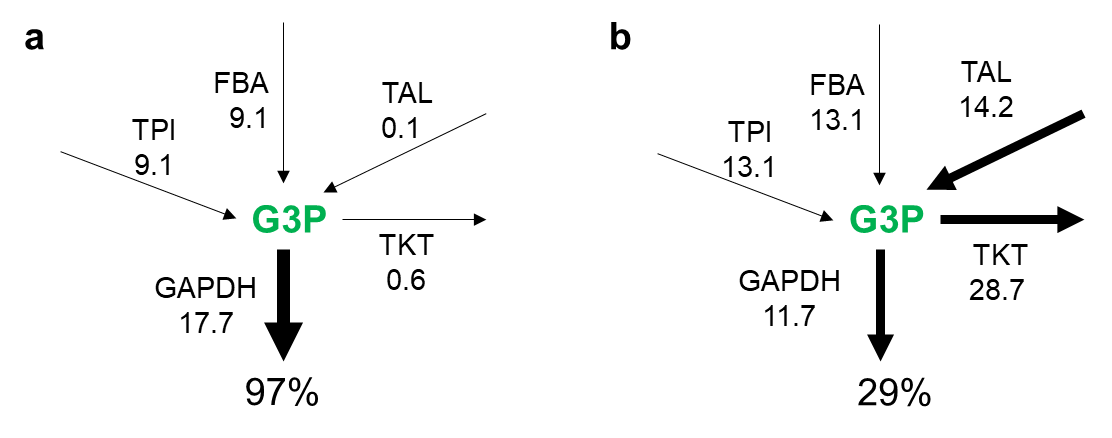


**Supplementary Figure 4. Simulated G3P fluxes during growth on glucose and methanol**

Fluxes were simulated using Flux Balance Analysis using the *E. coli* core model derived from iAF1260 and the Cobra Tool Box (Online Methods). **a**) Growth on glucose, during which 97% of the glyceraldehyde 3-phosphate (G3P) flux is channeled into lower glycolysis. **b**) Growth on methanol, during which the majority of G3P flux is into the pentose phosphate pathway to regenerate Ru5P. Additional abbreviations: FBA, fructose bisphosphate aldolase; TPI, triosephosphate isomerase; TAL, transaldolase; TKT, transketolase; GAPDH, glyceraldehyde-3-phosphate dehydrogenase.

**
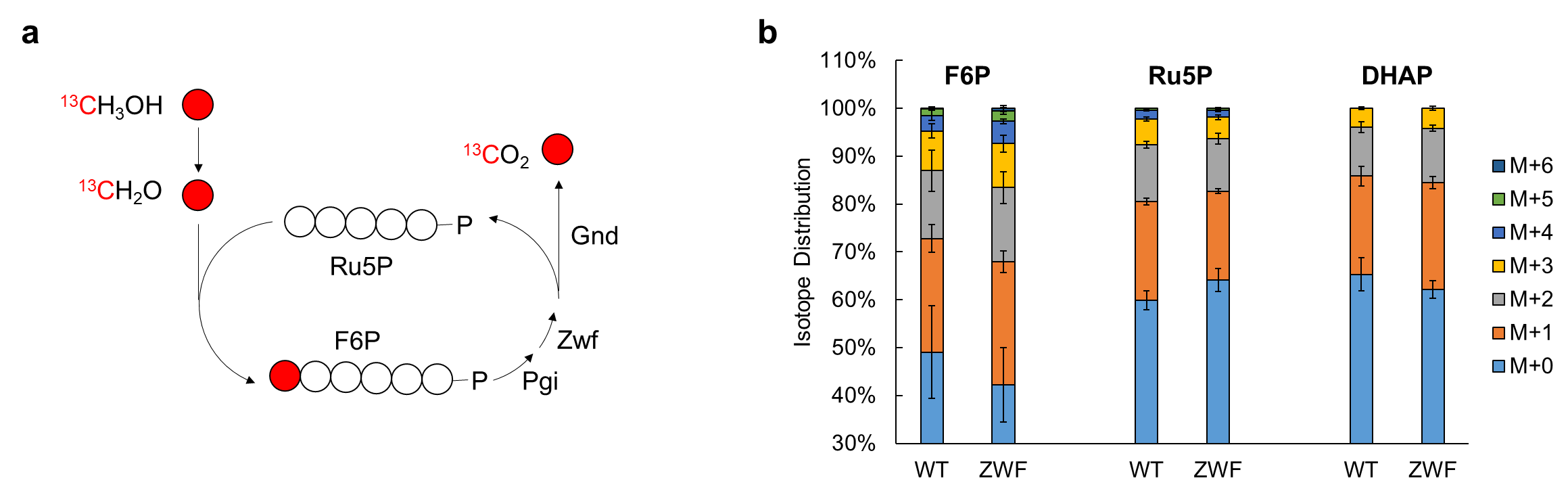
**

**Supplementary Figure 5. Oxidative PPP contribution to Ru5P is limited in resting cells**

**a**) Simplified pathway map showing conversion of fructose 6-phosphate (F6P) to ribulose 5-phosphate (Ru5P) through the oxidative pentose phosphate pathway, during which the labeled carbon (red) is lost as CO_2_, resulting in dissimilation of methanol. **b**) Isotopic enrichment of central metabolites was measured in resting cells of MG1655(DE3) ΔfrmA (WT), and MG1655(DE3) Δ*frmA* Δ*zwf* (ZWF), both carrying pETMEOH500, after treatment with 250 mM ^13^C methanol. The lack of difference in Ru5P labeling between the two cell lines indicates that the majority of Ru5P is provided through the non-oxidative PPP. The similarity in labeling of dihydroxyacetone phosphate (DHAP) between both strains indicates similar methanol assimilation, further confirming minimal flux through the oxidative PPP. Additional abbreviations: Pgi, phosphoglucose isomerase; Zwf, glucose 6-phosphate dehydrogenase; Gnd, 6-phosphogluconate dehydrogenase. Error bars represent s.d. of n = three biological replicates (individual colonies)

**
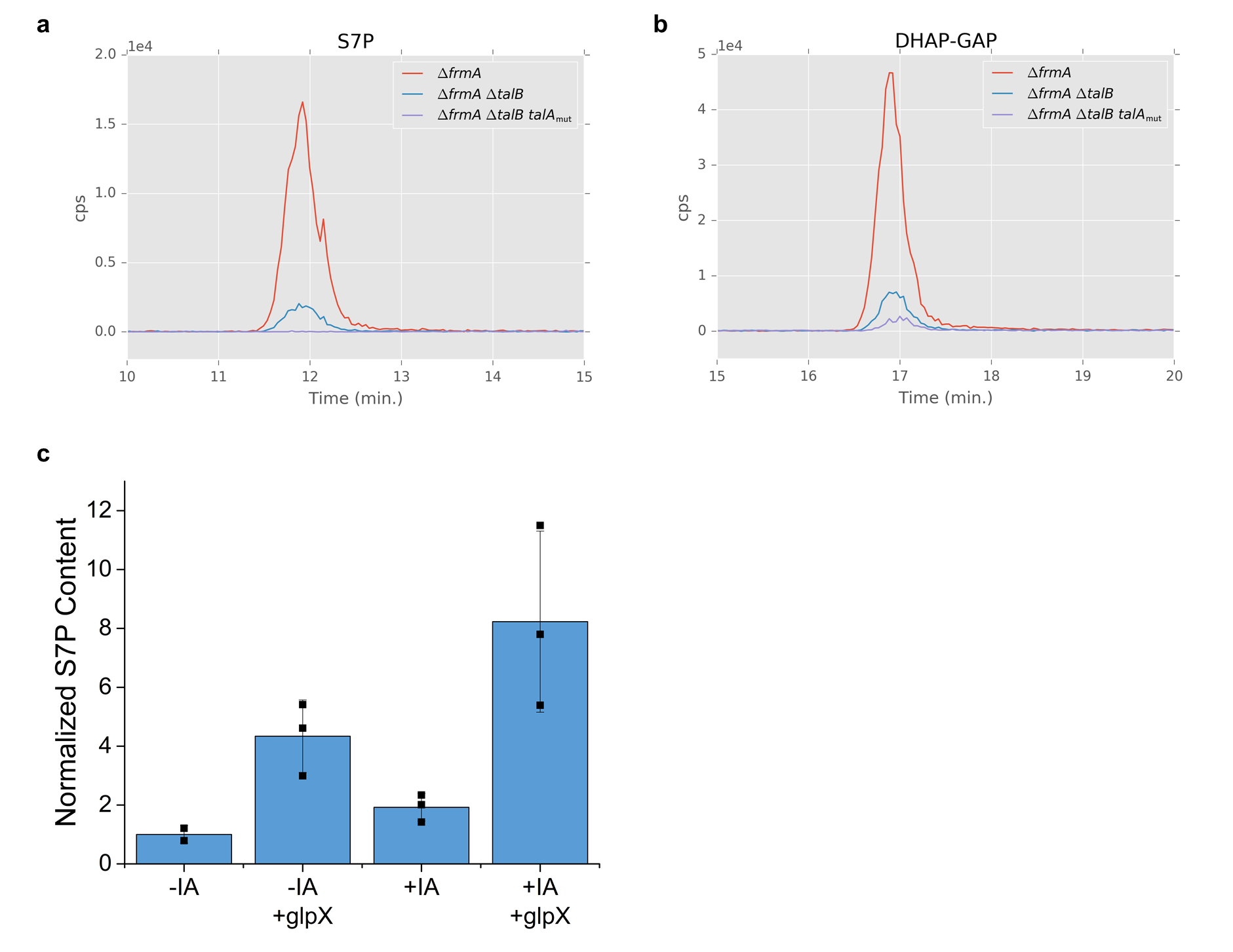
**

**Supplementary Figure 6. Enzymatic and metabolite measurements in transaldolase-deficient strain**

LC-MS/MS traces of transaldolase products S7P (**a**) and DHAP (**b**) production in assays with crude lysates of various strains, as described in the **Online Methods**. The *ΔfrmA* strain produced substantial quantities of both products. The *ΔfrmA ΔtalB* strain produced substantially less, but still detectable quantities, confirming TalB as the primary transaldolase, but revealing that TalA is also active under these conditions. The *ΔfrmA ΔtalB talA_mut_* strain (in which TalA has the mutation K131A) produced no detectable S7P, indicating complete lack of Tal activity in this strain. The small amount of DHAP produced by this strain is likely due to residual conversion of F6P 🡪 FBP 🡪 DHAP + G3P. **c**) Measurement of S7P in resting cells of *ΔfrmA ΔtalB talA_mut_* containing the full methanol pathway, with or without iodoacetate (IA) treatment, and with or without glpX overexpression. Abbreviations: S7P, sedoheptulose 7-phosphate; DHAP, dihydroxyacetone phosphate; F6P, fructose 6-phosphate; FBP, fructose 1,6-bisphosphate. Error bars represent s.d. of n = three biological replicates (individual colonies)

**
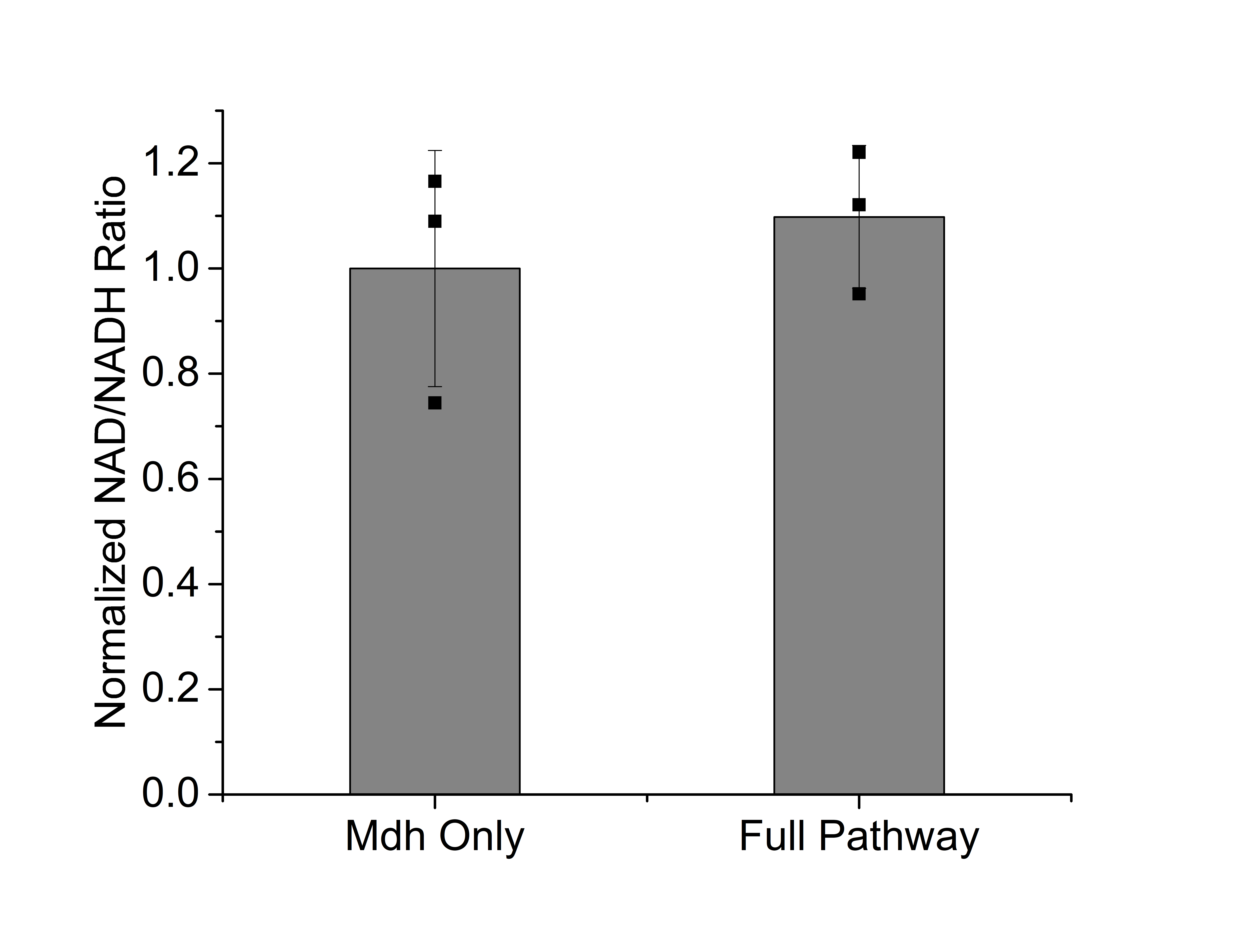
**

**Supplementary Figure 7. NAD/NADH ratio in cells expressing Mdh or full pathway**

NAD and NADH concentrations in resting cells of *E. coli* MG1655(DE3) *ΔfrmA* expressing Mdh only, or the full pathway (Mdh, Hps, Phi) after addition of 250 mM methanol were quantified by LC-MS/MS as described in the methods sections, and the ratios normalized to the Mdh case. No statistically significant difference was detected (2-tailed T-test, P=0.55, n=3 individual colonies). Error bars represent s.d. of n = three biological replicates (individual colonies)

**
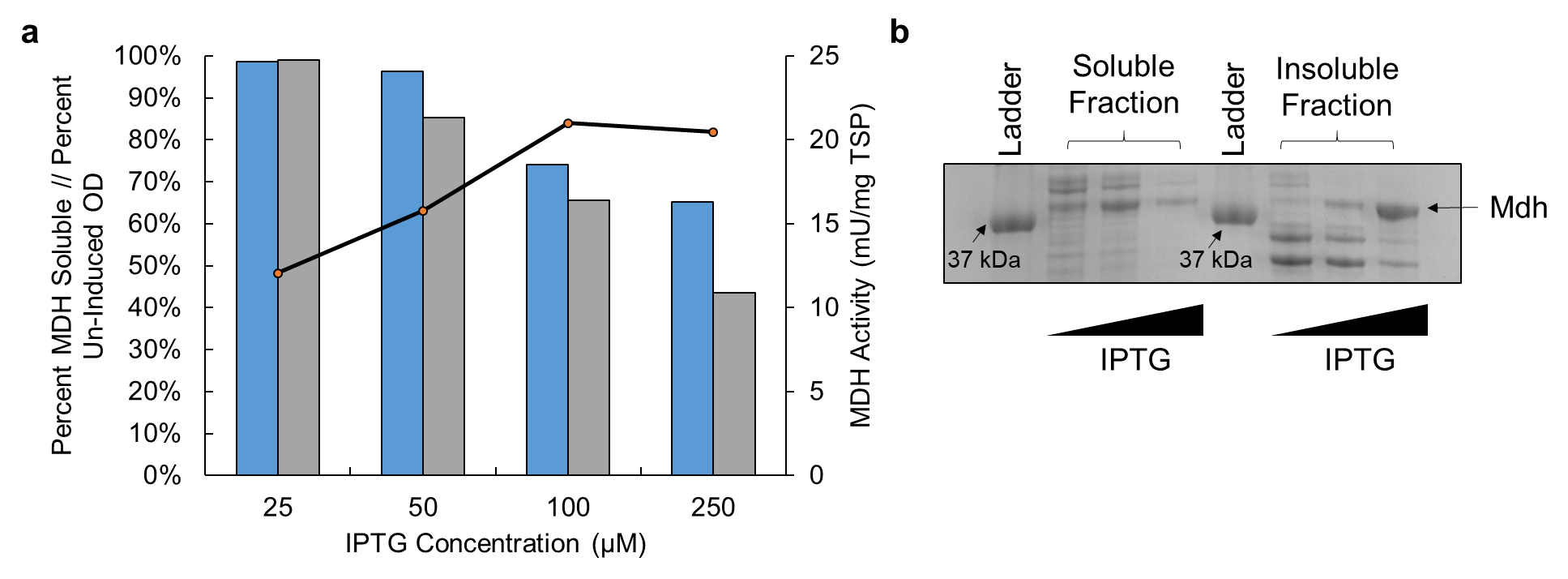
**

**Supplementary Figure 8. Effect of IPTG concentration on Mdh expression**

**a)** Effect of IPTG concentration on Mdh solubility, cell growth, and total Mdh activity. Blue bars: Percentage of total Mdh in soluble fraction. Gray bars: Culture OD 3 hours after induction, normalized to no-induction control. Black lines: Activity of Mdh in crude lysates. **b)** SDS-PAGE analysis of Mdh expression in crude lysates used to calculate percent solubility in a). Abbreviations: IPTG, isopropyl β-D-1-thiogalactopyranoside; Mdh, methanol dehydrogenase.

**
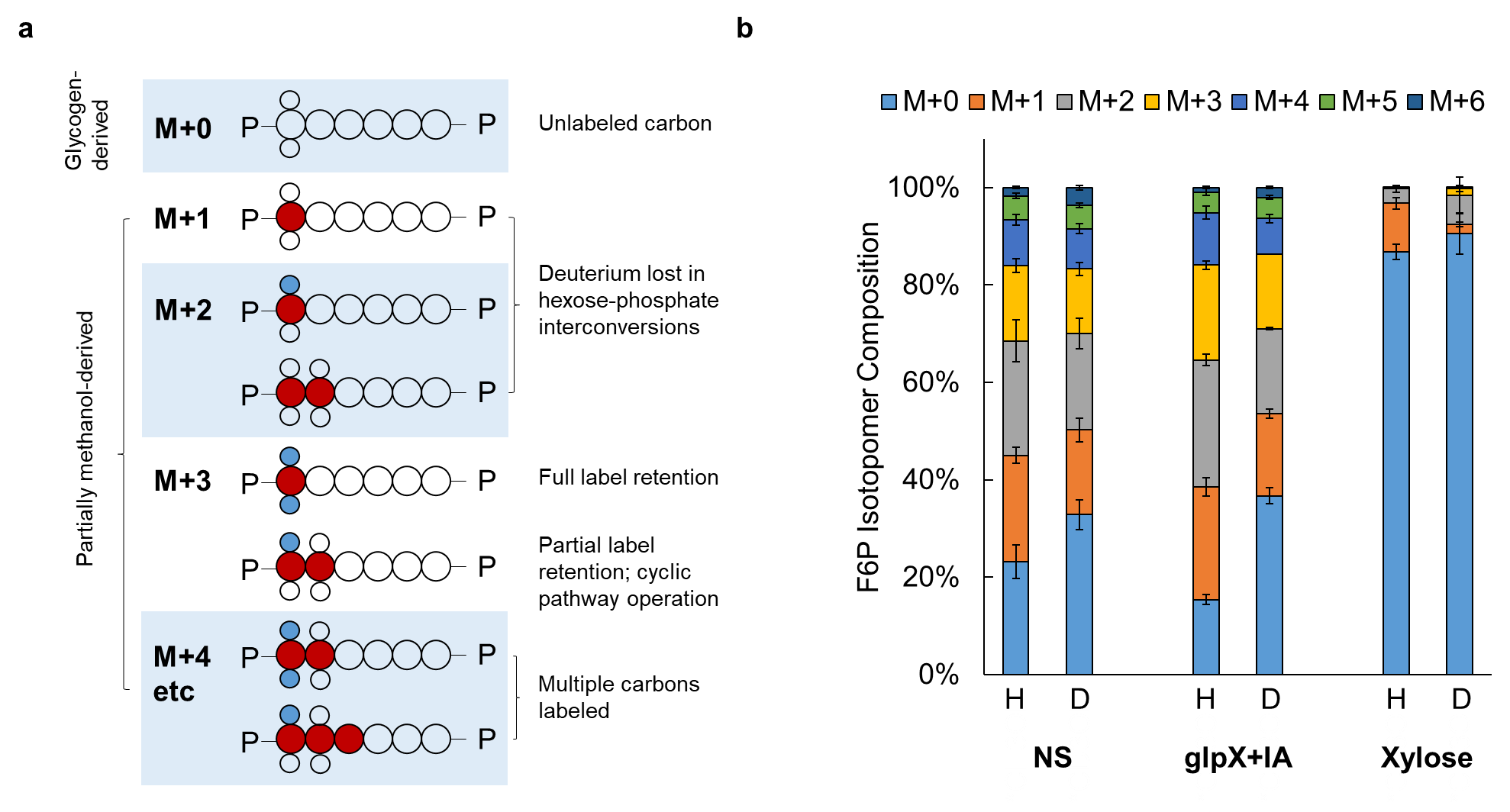
**

**Supplementary Figure 9. Isotopic labeling analysis of cells treated with deuterated methanol**

**a)** Expected labeling patterns from the metabolism of ^13^CD_3_OD. Quantitative flux analysis is prohibited by H-D exchange during hexose phosphate interconversions, but the ratio of fully unlabeled F6P (M+0) to the sum of all heavy isomers (M+1…M+6) is indicative of the rate of methanol assimilation flux. **b)** Isotopic analysis of fructose 6-phosphate (F6P) in cells treated with ^13^CD_3_OD. NS denotes no substrate, and MG1655(DE3) ΔfrmA carrying pETMEOH500. Xylose denotes the same strain, supplemented with 6 g L^-1^ xylose. glpX+IA represents the same strain but carrying both pETMEOH500 and pACglpX, and treated with 1 mM iodoacetate. All error bars represent s.d of n = 3 biological replicates (individual colonies).

**Supplementary Table 1. Heats of formation values for species in Mdh reaction**

| **Species** | ***H*_f_ (kJ mol^-1^)** | **Reference** |
| --- | --- | --- |
| CH_3_OH | -238.0 | NIST Webbook |
| CH_2_O | -109.0 | ^5^ |
| H_2_ | 0 | Definition |

**Supplementary Table 2. Kinetic parameters of methanol pathway enzymes**

| **Enzyme** | ***k*_cat_** | ***K*_m_** | **Reference** |
| --- | --- | --- | --- |
| MDH (*C. necator* v.4-1) | 0.2 s^-1^ | 21.6 mM (Methanol, pH 9.5) | ^1^ |
| HPS (*B. methanolicus*) | 24.7 s^-1^ | 147 μM (Formaldehyde)  450 μM (Ru5P) | ^2^ |
| PHI (*M. capsulatus*) | 493.1 s^-1^ | 100 μM | ^3^ |

**Supplementary Table 3. Strains and plasmids used in this study**

| **Name** | **Description** | **Reference** |
| --- | --- | --- |
| *Strains* | | |
| *E. coli* DH5α | Cloning strain | NEB |
| *E. coli* BL21(DE3) | Protein expression and purification | NEB |
| *E. coli* MG1655(DE3) | Background for methanol assimilation studies  F^-^ λ^-^ ilvG rfp-50 rph-1 (DE3) | ^6^ |
| *ΔfrmA* | MG1655(DE3) with *frmA* knocked out | This work |
| *ΔfrmA ΔtalB* | MG1655(DE3) with *frmA* and *talB* knocked out | This work |
| *ΔfrmA ΔtalB talA(K131A)* | MG1655(DE3) with *frmA* and *talB* knocked out, and K131A mutation in talA. Transaldolase-deficient strain | This work |
| *Plasmids* | | |
| pET28 | ColE1 lacI pT7 Kn^R^ | Novagen |
| pETmdh | pET28 with *C. necator* mdh2 4.1 | This work |
| pETHISmdh | pETmdh with N-terminal 6-HIS tag | This work |
| pETMEOH500 | pETmdh with hps from *B. methanolicus* and phi from *M. capsulatus* | This work |
| pETHISglpX | pET28 with *E. coli glpX* and N-terminal 6-HIS tag | This work |
| pETHISfbaA | pET28 with E. coli *fbaA* and N-terminal 6-HIS tag | This work |
| pACYC-Duet | P15a lacI pT7 Cm^R^ | Novagen |
| pACglpX | pACYC-Duet carrying *E. coli* *glpX* | This work |
| pTargetF | pMB1 aadA sgRNA-pMB1 | ^7^ |
| pCas9 | repA101(Ts) kan P_cas_-cas9 P_araB_-Red lacI^q^ P_trc_-sgRNA-pMB1 | ^7^ |
| pTarget-frmA | pTargetF with homology for *frmA* | This work |
| pTarget-frmA-N20 | pTargetF with homology and N20 for *frmA* | This work |
| pTarget-talB | pTargetF with homology for *talB* | This work |
| pTarget-talB-N20 | pTargetF with homology and N20 for *talB* | This work |
| pTarget-dtalA | pTargetF with homology for *talA* | This work |
| pTarget-dtalA-N20 | pTargetF with homology and N20 for *talA* | This work |

**Supplementary Table 4. Primers used in this study**

| **Fragment** | **Template** | **Primer** | **Sequence** |
| --- | --- | --- | --- |
| **pETmdh** | | | |
| *C. necator mdh2* | GBlock | mdh2_F  mdh2_R | gaaataattttgtttaactttaagaaggagatatacc**ATG**ACCCACCTGAACATCGCTAA  tgttagcagccggatctcaTTACATCGCCGCAGCGAAGATTG |
| Backbone | pET28 | pET28_R  pET28_F | GGTATATCTCCTTCTTAAAGTTAAAC  TGAGATCCGGCTGCTAACA |
| **pETHISmdh** | | | |
| *C. necator mdh2* | pETmdh | mdh2_HIS_F  mdh2_R | gaaataattttgtttaactttaagaaggagatatacc**atg**catcatcatcatcatcacacccacctgaacatcgctaa  tgttagcagccggatctcaTTACATCGCCGCAGCGAAGATTG |
| Backbone | pET28 | pET28_R  pET28_F | GGTATATCTCCTTCTTAAAGTTAAAC  TGAGATCCGGCTGCTAACA |
| **pETMEOH500** | | | |
| *C. necator mdh2* | GBlock | mdh2_F  mdh2_R_Path | ctttaagaaggagatatacc**ATG**ACCCACCTGAACATCGC  cctcgtttcttggttcttcgTTACATCGCCGCAGCGAAGATTG |
| *B. methanolicus hps* | GBlock | RBS-hps_F  hps-R-Path | cgaagaaccaagaaacgaggaggtcataaa**ATG**GAATTGCAATTAGCCTTAG  ggttaattcctcctggatccTTACAGACCTTGTTTTACAAGTTTATTAATC |
| *M. capsulatus phi* | GBlock | RBS-phi-F  phi-R | ggatccaggaggaattaacc**ATG**CATCAGAAACTCATTATC  gtcgacggagctcgaattcgTCATTCCAAATTAGCGTGAC |
| Backbone | pET28 | pET28_R  pET28_F-Path | GGTATATCTCCTTCTTAAAGTTAAAC  CGAATTCGAGCTCCGTCGAC |
| **pETHISglpX** | | | |
| *E. coli glpX* | gDNA | glpX_F  glpX_R | ctttaagaaggagatataccatgcatcatcatcatcatcacAGACGAGAACTTGCCATC  ttgttagcagccggatctcaTCAGAGGATGTGCACCTG |
| Backbone | pET28 | pET28_R-HIS  pET28_F | gtgatgatgatgatgatgcatGGTATATCTCCTTCTTAAAGTTAAACAAAATTATTTC  TGAGATCCGGCTGCTAAC |
| **pETHISfbaA** | | | |
| *E. coli fbaA* | gDNA | fbaA_F  fbaA_R | ctttaagaaggagatataccatgcatcatcatcatcatcacTCTAAGATTTTTGATTTCGTAAAAC  ttgttagcagccggatctcaTTACAGAACGTCGATCGC |
| Backbone | pET28 | pET28_R-HIS  pET28_F | gtgatgatgatgatgatgcatGGTATATCTCCTTCTTAAAGTTAAACAAAATTATTTC  TGAGATCCGGCTGCTAAC |
| **pACglpX** | | | |
| *E. coli glpX* | gDNA | glpX_F  glpX_R | ctttaataaggagatataccATGAGACGAGAACTTGCC  cagcggtttctttaccagacTCAGAGGATGTGCACCTG |
| Backbone | pACYC | pAC_R  pAC_F | GGTATATCTCCTTATTAAAGTTAAACAAAATTATTTCTACAGGG  GTCTGGTAAAGAAACCGCTG |
| **pTarget-frmA** | | | |
| Upstream homology | gDNA | frmA_UP_fwd  frmA_UP_rev | tattaccctgttatccctacTTCCTTCTGCCGCCCGCT  ttacggttcgCATCTCTCGCTCTTCCTCAATATGGTAATAGATTC |
| Downstream homology | gDNA | frmA_DN_fwd  frmA_DN_rev | gcgagagatgCGAACCGTAATTCGTTACTG  tgatggagctgcacatgaacACTAAATCCGGCAGCTCG |
| Backbone | pTarget-F | Digest (XhoI) | N/A |
| **pTarget-frmA-N20** | | | |
| Whole plasmid | pTarget-frmA | frmA-sg1-F  pTarget-sg-R | gtcctaggtataatactagtCTAATTAAAGTCACCCATACgttttagagctagaaatagc  ACTAGTATTATACCTAGGACTGAG |
| **pTarget-talB** | | | |
| Upstream homology | gDNA | talB_UP_fwd  talB_UP_rev | GAAGCTTAGATCTATTACCCTGTTATCCCTACACCCACGCGCGCCGCAAG  AATGATTACAGCAGATCGCCGATCATCATGATAGTATTTCTCTTTAAACAGCTTGTTAGG |
| Downstream homology | gDNA | talB_DN_fwd  talB_DN_rev | CTAACAAGCTGTTTAAAGAGAAATACTATCATGATGATCGGCGATCTGCTGTAATCATTC  CTGATGGAGCTGCACATGAACACGCCCACCTGACGCGAAAG |
| Backbone | pTarget-F | pT-BB-F  pT-BB-R | GTTCATGTGCAGCTCCATCAG  GTAGGGATAACAGGGTAATAGATCTAAGCTTC |
| **pTarget-talB-N20** | | | |
| Whole plasmid | pTarget-talB | talB-sg1-F  pTarget-sg-R | GTCCTAGGTATAATACTAGTCGCTCAGGCTCGTGCTTGTGGTTTTAGAGCTAGAAATAGC  ACTAGTATTATACCTAGGACTGAG |
| **pTarget-dtalA** | | | |
| Upstream homology | gDNA | dTalA-UP_fwd  dTalA-UP_rev | GAAGCTTAGATCTATTACCCTGTTATCCCTACATCACCGCCACCAGGTTACCTC  CCTTCCCAGGTCGAAGCCAGCGCGATCAGAATGCGTGATTTCTCAAC |
| Downstream homology | gDNA | dTalA-DN_fwd  dTalA-DN_rev | GTTGAGAAATCACGCATTCTGATCGCGCTGGCTTCGACCTGGGAAGG  CTGATGGAGCTGCACATGAACCTTCTTGCCGCCAAACTATAAAC |
| Backbone | pTarget-F | pT-BB-F  pT-BB-R | GTTCATGTGCAGCTCCATCAG  GTAGGGATAACAGGGTAATAGATCTAAGCTTC |
| **pTarget-dtalA-N20** | | | |
| Whole plasmid | pTarget-talB | dtalA-sg1-F  pTarget-sg-R | GTCCTAGGTATAATACTAGTATCACGCATTCTGATCAAGCGTTTTAGAGCTAGAAATAGC  ACTAGTATTATACCTAGGACTGAG |
| **pTarget-zwf** |  |  |  |
| Upstream homology | gDNA | zwf-UP_fwd  zwf-UP_rev | TATTACCCTGTTATCCCTACAACGATTCACCGTCGGTTC  CATTCCAGGACATGTCATTCTCCTTAAGTTAACTAAC |
| Downstream homology | gDNA | zwf-DN_fwd  zwf-DN_rev | GAATGACATGTCCTGGAATGAGTTTGAGTAATATCTGCG  TGATGGAGCTGCACATGAACTGGTGCGCGGAGAGCATG |
| Backbone | pTarget-F | pT-BB-F  pT-BB-R | GTTCATGTGCAGCTCCATCAG  GTAGGGATAACAGGGTAATAGATCTAAGCTTC |
| **pTarget-zwf-N20** |  |  |  |
| Whole plasmid | pTarget-talB | dtalA-sg1-F  pTarget-sg-R | GTCCTAGGTATAATACTAGTGCAGACGTTTACCAGTACGCGTTTTAGAGCTAGAAATAGC  ACTAGTATTATACCTAGGACTGAG |

**Supplementary Table 5. N20 sgRNA sequences used in this study**

| **Gene Target** | **N20 Sequence** |
| --- | --- |
| *frmA* | CTAATTAAAGTCACCCATAC |
| *talA* | GTATCACGCATTCTGATCAA |
| *talB* | CGCTCAGGCTCGTGCTTGTG |
| *zwf* | GCAGACGTTTACCAGTACGC |

**Supplementary Table 6. Kinetic parameters used in dynamic formaldehyde simulations^*^**

| **Mdh Parameters** | **Close to equilibrium** | | **Far from equilibrium** | |
| --- | --- | --- | --- | --- |
| *k*_1_ | 10 | 40 | 10 | 40 |
| *K*_eq_ | 0.05 | 0.05 | 0.05 | 0.05 |
| *k*_-1_ | 200 | 800 | 200 | 800 |
| [NAD] | 2 | 2 | 2 | 2 |
| [MeOH] | 10 | 10 | 10 | 10 |
| [NADH] | 0.1 | 0.1 | 0.1 | 0.1 |
|  |  |  |  |  |
| **Hps Parameters** |  |  |  |  |
| *k*_2_ | 2 | 2 | 100 | 100 |
| [Ru5P] | 1 | 1 | 1 | 1 |

^*^Governing Equation:

$\frac{d\left[ F \right]}{dt}=k_{1}\left[ \mathrm{NAD} \right] \left[ CH_{3}\mathrm{OH} \right]-k_{-1}\left[ \mathrm{NADH} \right]\left[ F \right]-k_{2}\left[ Ru5P \right]\left[ F \right]$ **(2)**

Where *k*_1_ and *k*_-1_ denote the forward and reverse second-order rate constants for Mdh, and *k*_2_ represents the irreversible second-order forward rate constant of Hps. All units are arbitrary. The equilibrium constant, *K*_eq_, is defined as *k*_1_/*k*_-1_.

The assimilatory flux is $k_{2}\left[ Ru5P \right]\left[ F \right]$, such that a higher steady state formaldehyde concentration reflects greater rate of methanol assimilation into metabolism.**Supplementary References**

1. Wu, T. Y. *et al.* Characterization and evolution of an activator-independent methanol dehydrogenase from Cupriavidus necator N-1. *Appl. Microbiol. Biotechnol.* **100,** 4969–4983 (2016).

2. Arfman, N., Bystrykh, L., Govorukhina, N. I. & Dijkhuizen, L. 3-Hexulose-6-phosphate synthase from thermotolerant methylotroph Bacillus C1. *Methods Enzymol.* **188,** 391–397 (1990).

3. Ferenci, T., Strom, T. & Quayle, J. R. Purification and properties of 3-hexulose phosphate synthase and phospho-3-hexuloisomerase from Methylococcus capsulatus. *Biochem. J.* **144,** 477–86 (1974).

4. Burton, K. The enthalpy change for the reduction of nicotinamide--adenine dinucleotide. *Biochem. J.* **143,** 365–368 (1974).

5. Da Silva, G., Bozzelli, J. W., Sebbar, N. & Bockhorn, H. Thermodynamic and Ab initio analysis of the controversial enthalpy of formation of formaldehyde. *ChemPhysChem* **7,** 1119–1126 (2006).

6. Tseng, H.-C., Martin, C. H., Nielsen, D. R. & Prather, K. L. J. Metabolic engineering of Escherichia coli for enhanced production of (R)- and (S)-3-hydroxybutyrate. *Appl. Environ. Microbiol.* **75,** 3137–45 (2009).

7. Jiang, Y. *et al.* Multigene Editing in the Escherichia coli Genome via the CRISPR-Cas9 System. *Appl. Environ. Microbiol.* **81,** 2506–2514 (2015).

8. Noor, E. *et al.* Pathway Thermodynamics Highlights Kinetic Obstacles in Central Metabolism. *PLoS Comput. Biol.* **10,** (2014).

9. Bennett, B. D. *et al.* Absolute metabolite concentrations and implied enzyme active site occupancy in Escherichia coli. *Nat. Chem. Biol.* **5,** 593–9 (2009).

10. Flamholz, A., Noor, E., Bar-Even, A. & Milo, R. EQuilibrator - The biochemical thermodynamics calculator. *Nucleic Acids Res.* **40,** 770–775 (2012).
