# Supplementary material for: Improving formaldehyde consumption drives methanol assimilation in engineered E. coli
Source: Nat Commun. 2018 Jun 19;9:2387. doi: 10.1038/s41467-018-04795-4 (PMC6008399; doi:10.1038/s41467-018-04795-4)
Supplement: Supplementary file 2 — Description of Additional Supplementary Files [file 41467_2018_4795_MOESM2_ESM.pdf]

Descriptions of Additional Supplementary Files

File Name: Supplementary Dataset 1
Description: Supplemental Dataset 1 contains the full simulated formaldehyde time course data that was used to generate Figure 6b.
